# Palladium Nanocubes as Saturable Absorbers for Mode-Locked Laser Generation at 1.56 μm

**DOI:** 10.3390/nano14231971

**Published:** 2024-12-08

**Authors:** Zhe Kang, Fang Wang

**Affiliations:** 1 Changchun Observatory of National Astronomical Observators, Chinese Academy of Sciences, Changchun 130117, China; kangz@cho.ac.cn; 2 College of Information Science and Engineering, Northeastern University, Shenyang 110819, China

**Keywords:** palladium (Pd) nanocubes, D-shaped fiber, mode-locked fiber laser

## Abstract

Palladium (Pd) nanocubes, a type of metallic nanostructure, have demonstrated remarkable optoelectronic properties, garnering significant attention. However, their nonlinear optical characteristics and related device applications remain underexplored. In this study, we report the fabrication of a novel saturable absorber (SA) by depositing Pd nanocubes onto a D-shaped fiber (DF). The Pd nanocubes, with an average size of 12 nm, were synthesized and integrated with a DF, resulting in a highly robust SA with broadband saturable absorption characteristics. When incorporated into Er^3+^-doped laser cavities, the Pd-DF SA enabled the generation of ultrafast pulses with a central wavelength of 1560 nm, a corresponding repetition rate of 26.7 MHz, and a temporal width of 1.85 ps. Our findings highlight the strong potential of the Pd-DF device as a versatile SA for constructing high-energy ultrafast fiber lasers.

## 1. Introduction

Over the past decade, mode-locked fiber lasers have gained significant attention and have been widely applied in areas such as spectroscopy, fiber sensing, material processing, and biomedical diagnostics. This is primarily due to their high-energy pulse output, cost-effectiveness, and compact design, among other advantages [1,2,3,4,5]. Traditionally, passive mode-locking, achieved using saturable absorbers (SAs), has been one of the most effective methods for generating ultrashort pulses in fiber lasers [6,7,8,9,10,11,12,13]. Among various SAs, semiconductor saturable absorber mirrors (SESAMs) are the most commonly utilized for constructing passively mode-locked fiber lasers due to their wideband operation wavelength. Moreover, the modulation depth, saturation flux, and unsaturated loss of the SESAMs can be controlled through structural design [14,15,16]. However, SESAMs are constrained by their narrow operational bandwidth (typically limited to a few tens of nanometers) and complex fabrication processes [17]. As a result, the development of novel SAs remains critical for advancing ultrafast photonics and expanding related applications.

Metal nanostructures have attracted extensive research interest due to their diverse applications in optics, electronics, information storage, and biological and chemical sensing [18,19,20,21,22]. Recently, metal nanomaterials, such as gold, silver, and copper, have been explored as potential SAs for mode-locked fiber lasers. Their appeal lies in their exceptional optical properties, including their high third-order nonlinearity, broad absorption spectra, and rapid response times, primarily driven by localized surface plasmon resonance (SPR) [23,24,25,26]. SPR is an optical phenomenon arising from the coherent oscillation of electron plasma at the surface of metallic nanoparticles. A key advantage of metal nanomaterials is the tunability of their SPR absorption peaks, which can be precisely controlled by adjusting the morphology and structural characteristics of the nanomaterials. In fact, the dependence of dielectric function on the wavelength/frequency also plays a key role. For example, how fast the optical properties vary with the wavelength can determine the LSPR peak shifts produced by changes in the local surrounding medium, establishing the refractive index sensitivity of the nanoparticles. Therefore, the relevance of the permittivity dependence on wavelength opens a new perspective to transition metals such as Pd as plasmonic counterparts to noble metals such as Au or Ag. Moreover, Pd as a third plasmonic material shows better properties in comparison with Au and Ag, for instance, higher stability at elevated temperatures for thermosplasmonic applications. Hence, Pd nanoparticles show potential in the construction of high-energy pulse lasers.

Palladium (Pd) nanoparticles, as a class of metal nanomaterials, have also received significant attention. They are widely used as primary catalysts for the low-temperature reduction of automotive pollutants and in various organic reactions, including Suzuki, Heck, and Stille couplings [27,28,29,30]. Generally, the catalytic performance of Pd nanoparticles can be optimized by controlling their particle size. Another important yet underexplored property of Pd nanoparticles is their surface plasmon resonance (SPR), which offers potential applications in colorimetric sensing, nanoscale waveguiding, electromagnetic field enhancement, and light transmission. These attributes suggest that Pd nanomaterials are promising candidates as saturable absorbers (SAs) for the generation of ultrashort optical pulses. However, the theoretical and experimental exploration of Pd nanomaterials in the field of ultrashort pulse generation remains at a preliminary stage. Therefore, there is a persistent drive to identify high-quality metal nanomaterials that exhibit nonlinear saturable absorption effects for efficient ultrashort pulse generation.

In our study, we report on passively mode-locked fiber lasers at 1560 nm by using a D-shaped fiber (DF) coated with Pd nanocubes as an SA for the first time. Pd nanocubes with an average size of 12 nm were synthesized and deposited onto a DF, resulting in a Pd-DF SA with high robustness and a pronounced saturable absorption response. The interaction between the propagating light and the Pd nanocubes introduced a strong evanescent field. When this SA was inserted into the laser cavity, ultrafast pulses operating at 1560 nm were achieved. Our experimental results confirm that the integrated Pd-DF device has significant potential as a nonlinear optical medium for high-performance optical applications. Our results indicate that the Pd-DF SA is a promising modulator with the potential for important applications in the field of ultrafast lasers.

## 2. Characterization of Pd Nanocubes

**Figure 1:**
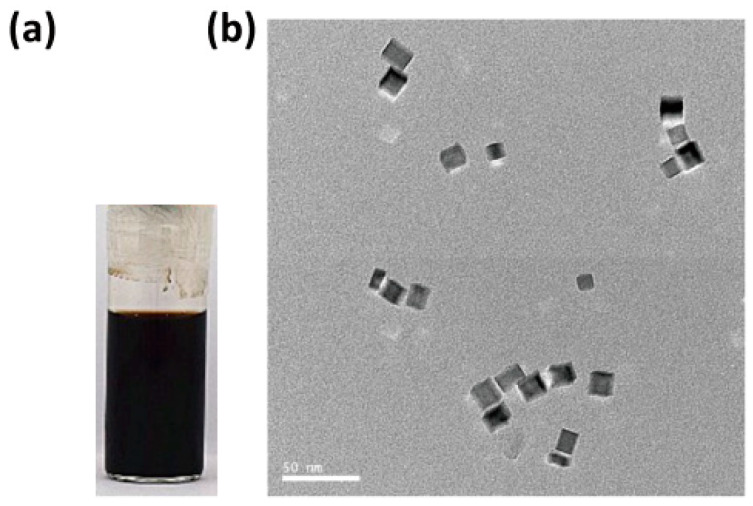
(**a**) Solution and (**b**) TEM image of the as-synthesized Pd nanocubes.

The Pd nanocubes were synthesized via the reduction of dihydrogen tetrachloropalladate (H_2_PdCl_4_) using sodium citrate as a reducing agent, with polyvinyl pyrrolidone (PVP) serving as a surfactant to stabilize the nanoparticle surfaces [29]. The resulting solution exhibited a brown–black color, as illustrated in Figure 1a. Transmission electron microscopy (TEM) images were obtained using an FEI Tecnai G2 F20 microscope operating at 200 kV (JEM-2010, JEOL, Tokyo, Japan), with a scale bar of 50 nm, as shown in Figure 1b. The average diameter of the Pd nanocubes was approximately 12 nm.

**Figure 2:**
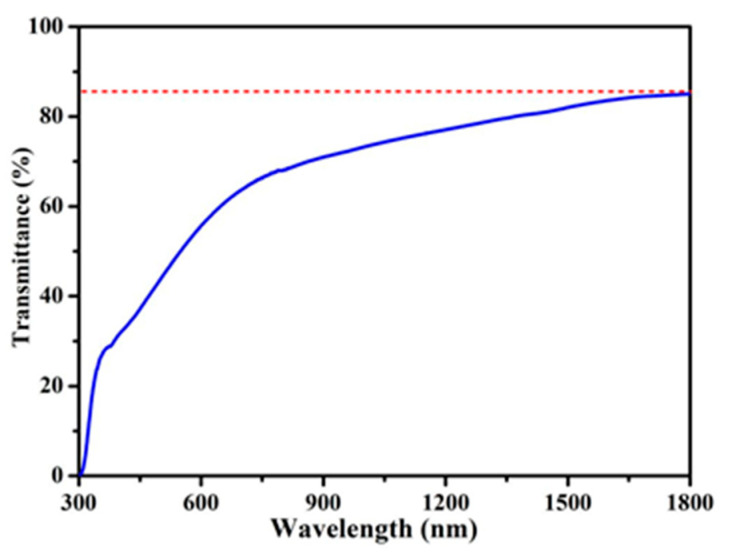
Absorption spectrum of the Pd nanocube film.

To examine the absorption characteristics at the Er^3+^ laser emission wavelength (1.56 μm), the absorption spectrum of the Pd nanocubes was measured using a film sample. This approach was chosen to avoid the strong absorption of liquid water typically observed between 1.4 and 1.8 μm. Figure 2 presents the absorption spectra of the Pd nanocube film, recorded with a UV-visible spectrophotometer (Agilent, Cary 5000, Santa Clara, CA, USA). The film exhibits broad absorption across a wide range, from 300 nm to 1800 nm, with a transmittance of approximately 80% at 1560 nm.

## 3. Preparation of the Pd-DF SA

**Figure 3:**
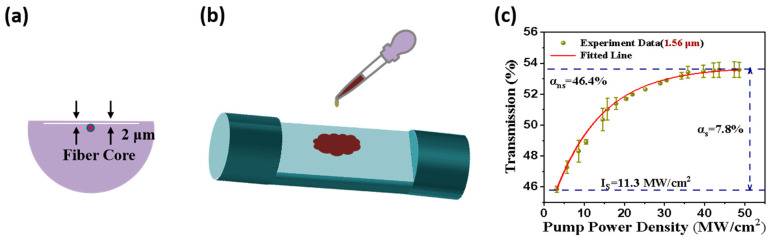
(**a**) DF structure. (**b**) Fabrication process. (**c**) Nonlinear transmission response at 1.56 μm.

To obtain high-performance pulses, Pd nanocubes were combined with DFs to prepare the SA. Compared to film-based and microfiber SAs, DF-based SAs offer a higher damage threshold, enabling longer nonlinear interaction lengths. Additionally, DFs demonstrate excellent robustness and can be reused. The DF was manufactured using an ultraprecision side-polished method. With a 2 μm distance between the D-shaped surface and the fiber core (Figure 3a), a small amount of aqueous Pd nanocube solution was applied to the polished surface to form the SA (Figure 3b). To further assess its potential in mode-locked fiber lasers, the nonlinear absorption properties of the integrated modulator were examined. The transmission behavior of the SA was measured using pulsed lasers operating at 1.56 μm. As shown in Figure 3c, the transmission ratio varied with changes in peak power density. The data were fitted using an equation, α(I) = α_s_/(1 + I/I_s_)+ α_ns_, from reference [31], where α(I) is the absorption coefficient and α_s_ and α_ns_ are the saturable and non-saturable absorption parameters, respectively, clearly revealing the saturable absorption properties. The modulation depth α_s_ and saturable intensity Is were determined to be 7.8% and 11.3 MW/cm^2^, respectively. These observations indicate that the Pd-DF SA presents the ability to generate an ultrafast pulse at 1.56 μm.

## 4. Results and Discussion

**Figure 4:**
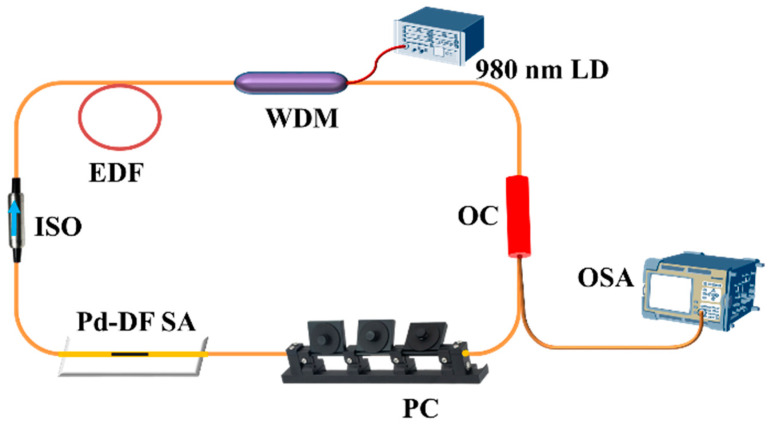
Schematic diagram of the mode-locked EDFL.

The structure of the EDFL is illustrated in Figure 4. A 980 nm laser diode (LD) serves as the light source, with a 980/1550 nm wavelength division multiplexer (WDM) directing the beam into the ring cavity. The system employs a 20 cm erbium-doped fiber (EDF) as the gain medium. An isolator (ISO) is incorporated to ensure unidirectional light propagation. The saturable absorber (SA) modulates the phase of the signal light, enabling a fixed phase difference essential for mode-locking. To maintain stable mode-locking, a polarization controller (PC) is included to adjust the polarization state within the cavity. Additionally, a 10% output coupler (OC) is used to extract a portion of the signal for analysis. The spectral characteristics and pulse sequences are monitored using an optical spectrum analyzer (OSA, Yokogawa AQ6375B) and a digital oscilloscope (Tektronix MDO3052), respectively.

**Figure 5:**
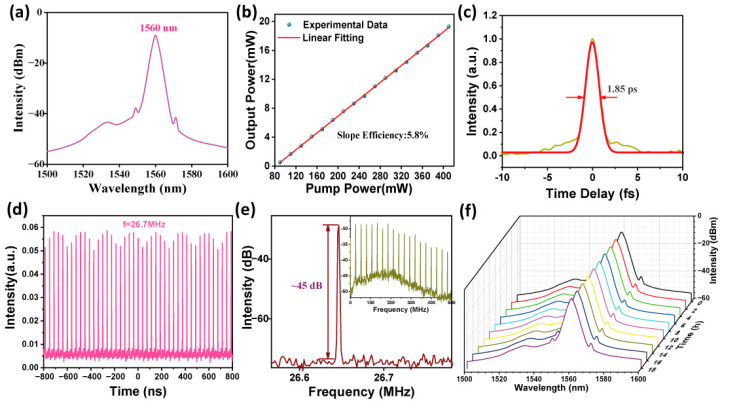
(**a**) Spectrum. (**b**) Power variation. (**c**) Pulse width. (**d**) Pulse sequence. (**e**) RF property. (**f**) Stability test.

For verification, we experimentally confirm the performance of the Pd-DF SA for mode-locked modulation. Furthermore, achieving stable mode-locking at 1560 nm is a highly worthwhile endeavor for optical communication and frequency conversion. The Pd-DF modulator was integrated into a custom-built erbium-doped fiber laser (EDFL). By optimizing the PC settings and gradually increasing the LD power, pulses were achieved at 90 mW. When the pump power was further increased to 410 mW, stable mode-locked operation was sustained at 1560 nm, as shown in Figure 5a. The corresponding repetition rate was 26.7 MHz (Figure 5d), and the temporal width of individual pulses was 1.85 ps, exhibiting a Sech^2^ pulse profile after amplification (Figure 5c). The output power increased linearly from 0.52 mW to 19.28 mW, demonstrating a conversion efficiency of 5.8% (Figure 5b). The radio frequency (RF) spectrum (Figure 5e) showed a fundamental peak at 26.7 MHz with a high SNR of 45 dB. The inset in Figure 5e displays a wideband RF spectrum extending up to 500 MHz, confirming the stability of the output pulses and the single-pulse operation. The output spectra (Figure 5f) were continuously monitored over 18 h, with no significant shifts in the central wavelength observed. These results confirm the high-performance mode-locking capability of the EDFL modulated by the Pd-DF SA, demonstrating its potential for reliable ultrafast pulse generation.

**Figure 6:**
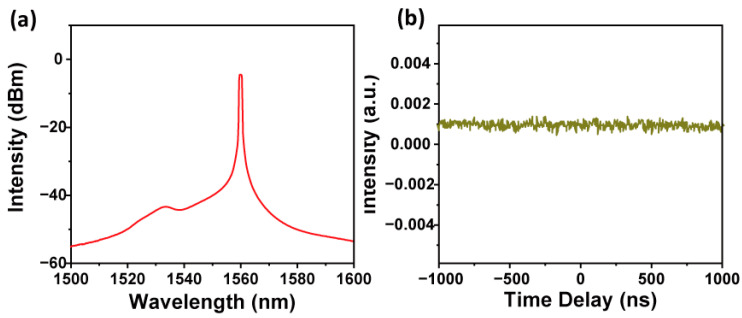
(**a**) Spectral and (**b**) temporal properties at 410 mW.

To further confirm the role of Pd nanocubes in enabling pulse generation, a bare DF was placed in the same all-fiber cavity. Despite adjustments in the PC and variations in the LD, no pulsed condition was obtained in the laser cavity. Figure 6 illustrates the spectral and temporal properties at 410 mW, clearly showing the absence of pulse generation when using a bare DF. This comparative result provides strong evidence that the mode-locked pulses were indeed modulated by the presence of Pd nanocubes.

In all, a novel nonlinear modulator was successfully prepared and verified based on the deposition of Pd nanocubes onto the DF. Compared with SAs based on metal nanoparticles [11,32,33,34], the Pd-DF SA not only presented better thermal stability (>410 mW) but also possessed higher output power (19.28 mW). Moreover, the generated pulses showed a higher SNR. The size of the Pd could be smartly controlled by adjusting the synthesis temperature and reaction time. By regulating the Pd SA and fiber cavity, the ability of the mode-locked operation would be significantly enhanced.

## 5. Conclusions

In conclusion, we successfully developed and validated a novel nonlinear modulator based on the deposition of Pd nanocubes onto the DF. Using this Pd-DF SA, we achieved ultrafast pulse generation at 1560 nm. The Pd nanocubes, with an average size of 12 nm, were synthesized and integrated with the DF, resulting in a modulator that exhibited broadband linear and nonlinear saturable absorption responses. When incorporated into laser cavities, this modulator enabled the realization of high-performance mode-locked fiber lasers. Our work introduces a promising broadband nonlinear modulator with potential for practical applications in ultrafast optoelectronics.

## Data Availability

Data are contained within the article.
